# Histologic description of acquired cholesteatomas: comparison between children and adults

**DOI:** 10.1016/S1808-8694(15)31020-X

**Published:** 2015-10-19

**Authors:** Cristina Dornelles, Luíse Meurer, Sady Selaimen da Costa, Cláudia Schweiger

**Affiliations:** a MS in Medical Sciences, PhD of the Post Graduation Program in Medical Sciences: Pediatrician of the Federal University of Rio Grande do Sul, Biologist from the Brazilian Center of Otitis Media, University Hospital of Porto Alegre – Department of Ophthalmology and Otorhinolaryngology - UFRGS; b PhD in Gastroenterology, Substitute Professor.; c PhD in Surgery, Associate Professor.; d 2nd year resident of Otorhinolaryngology Porto Alegre University Hospital - Federal University of Rio Grande do Sul.

**Keywords:** Cholesteatoma, Perimatrix, Inflammation

## Abstract

Cholesteatoma is constituted of matrix, perimatrix and cystic content. Some authors affirm that, in children, its clinical behavior is more aggressive of the than in adults.

**Aims:**

Histologic compared cholesteatomas of children and adults.

**Methodology:**

74 cholesteatomas been analyzed, being 35 of pediatrics patients (<18 years). The average number of cellular layers and hyperplasia in the matrix had been evaluated; thickness, delimitante epithelium, fibrosis, inflammation and granuloma in the perimatrix. The analysis statistics was carried through with program SPSS 10,0, using the coefficients of Pearson and Spearman, test of qui-square and t test. The number of cellular layers in the matrix was of 8,2±4,2. The hyperplasia appears in 17%, fibrosis in 65%, granuloma in 12% and the delimitante epithelium in 21%. The perimatrix presented a medium one of 80 micrometers (37 the 232), minimum value zero and maximum value 1.926. The histological degree of inflammation was considered of moderate the accented one in 60%. When applying the coefficient of Spearman enters the inflammation degree and average of cellular layers of the matrix with the variables of the measure of thickness of the perimatrix we find correlations, significant, with moderate magnitudes of the great ones (rs=0,5 and P<0,0001).

**Conclusion:**

Adults colesteatomas of and child had not been identified to morphologic differences between. We find correlation enters the intensity of the inflammation and of the average of cellular layers of the matrix with the thickness of the perimatrix, what it can predict its aggressiveness, more studies are necessary to define the paper of this finding in pathogenesis of cholesteatoma.

## INTRODUCTION

In chronic cholesteatomatous otitis media the lack of control over cell proliferation causes the growth of an epidermoid cyst, the cholesteatoma^1^, characterized by the presence of a keratinized stratified squamous epithelium inside any air-filled area of the temporal bone^2^. It is histologically made up of a matrix (epithelium), a perimatrix (sub-epithelial conjunctive tissue) and a cystic content (Keratin lamellae)^3^. Cholesteatomas may be considered a cell growth disorder, encompassing a number of complex and dynamic events, which involve cellular and extracellular components; its growth requires angiogenesis in the perimatrix connective tissue, and the substances present in the healing cascade may play an important role in its growth and development^4^. However, it is still unknown whether this lack of control is caused by defects in genes that control proliferation, by the cytokines released by inflammatory cells or by other, still unknown, mechanisms^5^. Thus, to determine defects in its biology, biochemistry and genetics it is critical to understand its pathogenesis.

Cholesteatomas have great erosion potential, they may reach the ossicular chain, skull bones and also the hardest bone in the human body, the labyrinth, - and this shows its important destructive capacity on the bone tissue. Total or partial ossicle destruction is seen in about 80% of the patients with cholesteatomas, while non-cholesteatomatous chronic otitis media causes ossicular chain erosion in only 20% of the cases^6^. The mechanisms that cause this increase in bone degradation and invasion are still under investigation^5^.

This pathology may affect children and adults alike, however, there are controversies shown in the literature as to its clinical manifestations in the different age ranges. Sheehy^7^, Tos^8^ and Edelstein^9^ believe the infantile cholesteatoma could be less expansive, and this would cause fewer complications. On the other hand, Galsscock^10^, Ruah^11^, Bujia^12^, Palva^13^ and Sudhoff^14^ believe the acquired cholesteatoma in children could be more aggressive and with more extensive growth. On the extreme of this disagreement, we find Smythe et al.^15^ believing that the clinical differences between children and adult are so large that they should be considered totally different diseases.

In an attempt to check if in children the clinical behavior of cholesteatomas depend on the histo-morphological characteristics of the perimatrix, Quaranta et al.^16^ carried out a study with samples taken from 30 patients below 16 years and 30 adults. Results show that in children, the perimatrix is rich in mononuclear inflammatory elements. The persistence of the inflammation could cause a permanent process of healing on the perimatrix, and such process would case the proliferation of fibroblasts (granulation tissue) and epithelium^6^. Based on such behavior, the authors concluded that the perimatrix characteristics could play an important role in the pathogenesis of the cholesteatoma and suggested that this would explain the clinical differences regarding cholesteatomas between children and adult patients^16^.

Many could have been the factors and chemical mediators involved in the aggressiveness and bone erosion behavior of cholesteatomas; however, if they were produced by the perimatrix, we could be able to determine the aggressiveness of a cholesteatoma thorough the analysis of its histological constitution, specially that of the perimatrix. Thus, this paper aims at histologically describing the cholesteatoma perimatrix acquired during childhood and adulthood, seen at light microscopy, as well as correlating such findings to the patient's age at the time of the surgery

## METHOD

This study was approved as far as its work ethics and methodologies are concerned, by the Group of Ethics in Research and Post-graduation of the HCPA, in 2002. All the patients who accepted to participate in the present study signed a Free Informed Consent Form, allowing us an anonymous use of this data in scientific publications and in the documentation and filing of the videotaping that was done. The fact that we did not obtain an informed consent form did not change the treatment we offered our patients. Our methodology is based on a contemporary, comparative and transversal study.

The patients included came from the Chronic Otitis Media Outpatient Ward of the University Hospital of Porto Alegre (AOMC-HCPA).

Patient inclusion in this study obeyed the following criteria:

1. Diagnosis of Cholesteatomatous Chronic Otitis Media;
2. Matrix and perimatrix presence in the cholesteatoma collected.

And the exclusion was made by a diagnosis of congenital cholesteatoma.

We studied 90 cholesteatomas, collected from otologic surgeries between May of 2003 and July of 2004, being 43 of those from pediatric patients (0 to 18 years) and 47 from adults (above 18 years).

**Figure 1:**
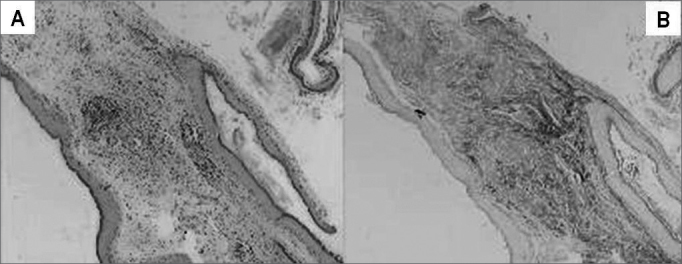
In this image we can see the same sample that was dyed with HE (A) and Sirius Red (B). Notice that the contrast obtained in the second dye is much greater, because the collagen fibers absorb better the Bordeaux red, and this enhances the thickness difference to be measured.

The material was collected by the otological surgeon, immediately fixed in 10% formaldehyde and processed by routine histological techniques, with inclusion in paraffin. We prepared two slides for morphological analysis of each sample. The slides were dyed by Hematoxylin-Eosin (HE) and Sirius Red, and analyzed under the light microscopy (Figure 1).

Notice on Figure 1 that the contrast obtained from the second dyeing is much greater, since in it the collagen fibers dyed in Bordeaux red, and this facilitates the differentiation of the thickness to be measured.

The material was read in a “blind” way and controlled by the researcher. The following components were observed: average number of cell layers and the presence of matrix hyperplasia; perimatrix thickness and outlining epithelium; fibrosis, perimatrix inflammation and granuloma.

The average number of matrix cell layers was obtained by counting them in five different fields on the slide and collecting them on a continuous fashion. For the inflammation histological grade we created an ordinal value from zero to three, zero = absent; 1 = mild, 2 = moderate and 3 = intense; being characterized by how much the perimatrix is crossed by lymphocytes, neutrophils, plasmocytes and macrophages^17^. The remaining variables, matrix hyperplasia, perimatrix thickness and outlining epithelium, fibrosis, granuloma and inflammation on the perimatrix, were collected on a qualitative basis, classified either as present or absent.

**Figure 2:**
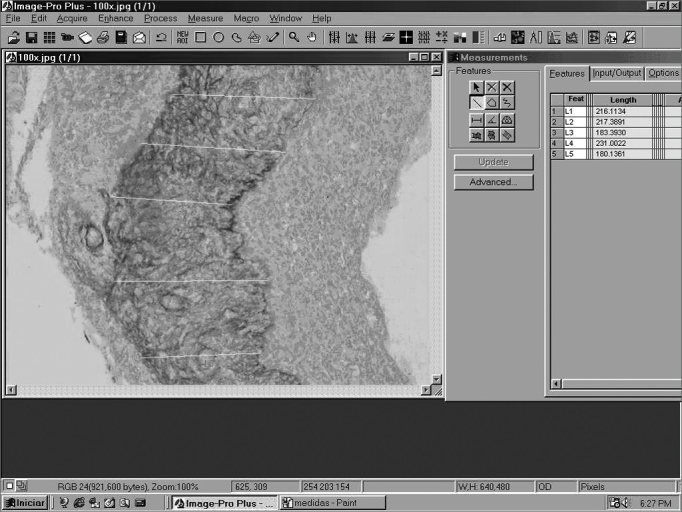
Image of the ImagePro Plus Media Cybernetics software screen.

The perimatrix thickness was obtained through the analysis of computerized images by means of the ImagePro Plus Media Cybernetics (Figure 2) image software. From each sample we used 20 measures of perimatrix thickness, which were summarized in average, median, minimum size, maximum size, summation and delta (maximum minus minimum), and these were the parameters used to test the correlation between perimatrix thickness with patient's age at the date of surgery.

For the statistical analysis we used both the Pearson and the Spearman coefficients in order to analyze the correlation between the average number of cell layers on the perimatrix and the histological grade of inflammation with the perimatrix thickness. For the descriptive data we used frequency tables, and the comparison between the groups was carried out by the c^2^ and the Student t tests, using the SPSS 10.0 for Windows software. P values below 0.05 were considered statistically significant.

## RESULTS

### Epidemiological Data

The sample counted on 86 patients, nine of them with bilateral pathology (six children and three adults), making up a total of 95 cholesteatomas; however, 21 were taken off the analysis since their slides presented only corneal lamellae.

In the group of excluded patients we had nine with age up to 18 years, with mean ± standard deviation equal to 12.74 ± 3.59 years. With more than 18 years there were 12 patients, with mean age ± standard deviation equal to 39.75 ± 12.87 years. As to gender, 67% were males - 44% in the pediatric group and 83% were adults.

Of the 74 patients included, mean age ± standard deviation was of 25.66 ± 16.31. In this group we had 35 with age up to 18 years, with mean age ± standard deviation equal to 12.85 ± 3.63 years. There were 39 patients with more than 18 years of age, with mean age ± standard deviation equal to 36.78 ± 14.68 years. As to gender, 48% of the sample was made up of males. Among children, this percentage was of 52%, now, among adults it was of 44%.

### Histology

**Figure 3:**
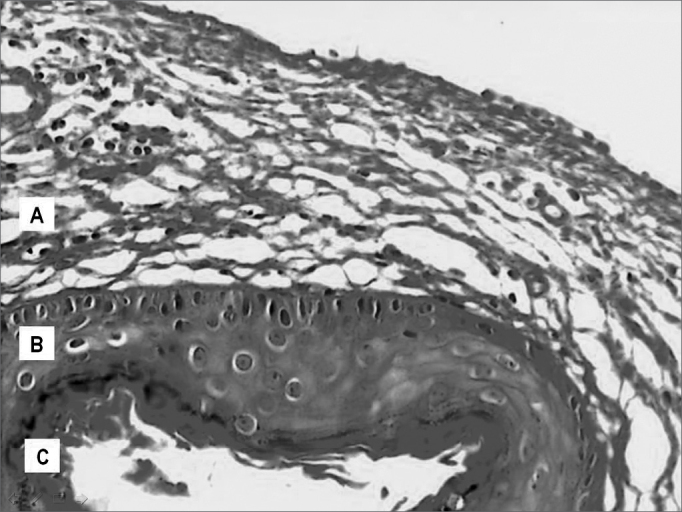
Digitalized image of the slide with a cholesteatoma cross-section, dyed in Hematoxylin-Eosin; presenting its constituting parts: A. Perimatrix; B. Matrix; C. Cystic content.

According to descriptions found in the literature, the cholesteatomas included in this sample, seen through light microscopy have a cystic formation covered by keratinized, stratified squamous epithelium, the so called matrix, laying on top of a dense connective tissue of varied thickness - the perimatrix. These irregularities appear as we see cholesteatomas coming from different patients, as well as in the material taken from the same individual. The same surgical specimen presented regions of different thicknesses, from very delicate to very thick. Often times the perimatrix presented lympho-plasmocytic infiltrate and/or granulation tissue and foreign body type of reaction, specially if there were lesion rupture. The cystic content was made up of keratin lamellae. Figure 3 shows an example that does represent well the histologic make up of acquired cholesteatomas.

We may state that the cholesteatoma coating (matrix) is undistinguishable from that of a squamous mucosa (squamous stratified epithelium, with an underlying corium - lamina propria, as the ones found in the mouth, vagina and esophagus) and the skin, except for the fact that the latter has skin annexes (hair and glands). They may also not be distinguished from epidermal or infundibular cysts for any site.

**Figure 4:**
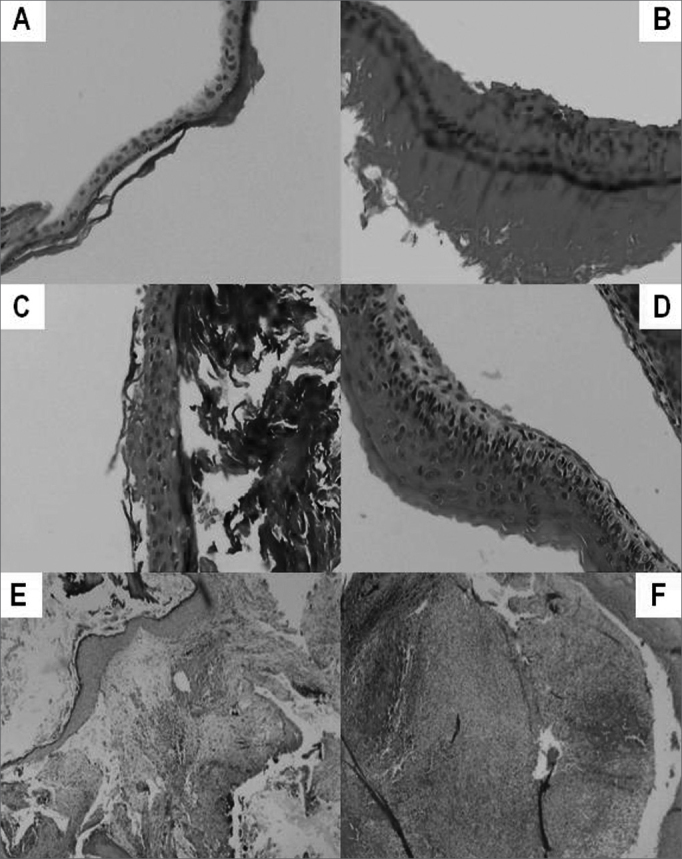
**A** - Stratified squamous epithelium, keratinized, with an average of three cell layers. No perimatrix. **B** - Stratified squamous epithelium, keratinized, with an average of six cell layers. Thin perimatrix, fibrotic, with rare lymphocytes. No granulomas. **C** Stratified squamous epithelium, keratinized, with an average of four cell layers. Very thin perimatrix, without fibrosis and without inflammatory infiltrate. **D** Stratified squamous epithelium, keratinized, with an average of six cell layers – Thin and delicate perimatrix, without fibrosis and with mild inflammatory infiltrate. **E** - Stratified squamous epithelium, keratinized, with an average of twelve cell layers. Perimatrix has dense fibrosis, chronic and intense inflammatory infiltrate and is deeply outlined by a simple cuboid epithelium. **F** - Stratified squamous epithelium, keratinized, with an average of thirteen cell layers with epithelial hyperplasia. Perimatrix with mild fibrosis with intense inflammatory infiltrate and neutrocyte exudation, deeply outlined by a simple cuboid epithelium. No granulomas.

Figure 4, shows some cross section images of acquired cholesteatomas, showing the great variability in perimatrix thickness, as well as its histology components.

The number of layers of the squamous epithelium varied between 3 and 23 (7.94 ± 4.15). When stratified by age range there is not much modification, 7.87 ± 4.22 (minimum = 3 and maximum = 22), in the pediatric group, and 8.00 ± 4.15 (minimum = 4 and maximum = 23), in the adult group (P=0.89).

The perimatrix appears as an inflammatory network that surrounds the cholesteatoma, of variable thickness, both intra and inter-patient. The total group presented a median value of 80 micrometer with inter-quartile interval of 37 to 232, having zero as the minimum value and 1,926 as its maximum. When the perimatrix thickness median was stratified by age of presentation, in the pediatric group, a median of 79 (41 to 259), with minimum value equal to zero and maximum equal to 484; and in the adult group, a median of 83 (26 to 174), having zero as the minimum value and 1,926 as maximum value.

**Figure 5:**
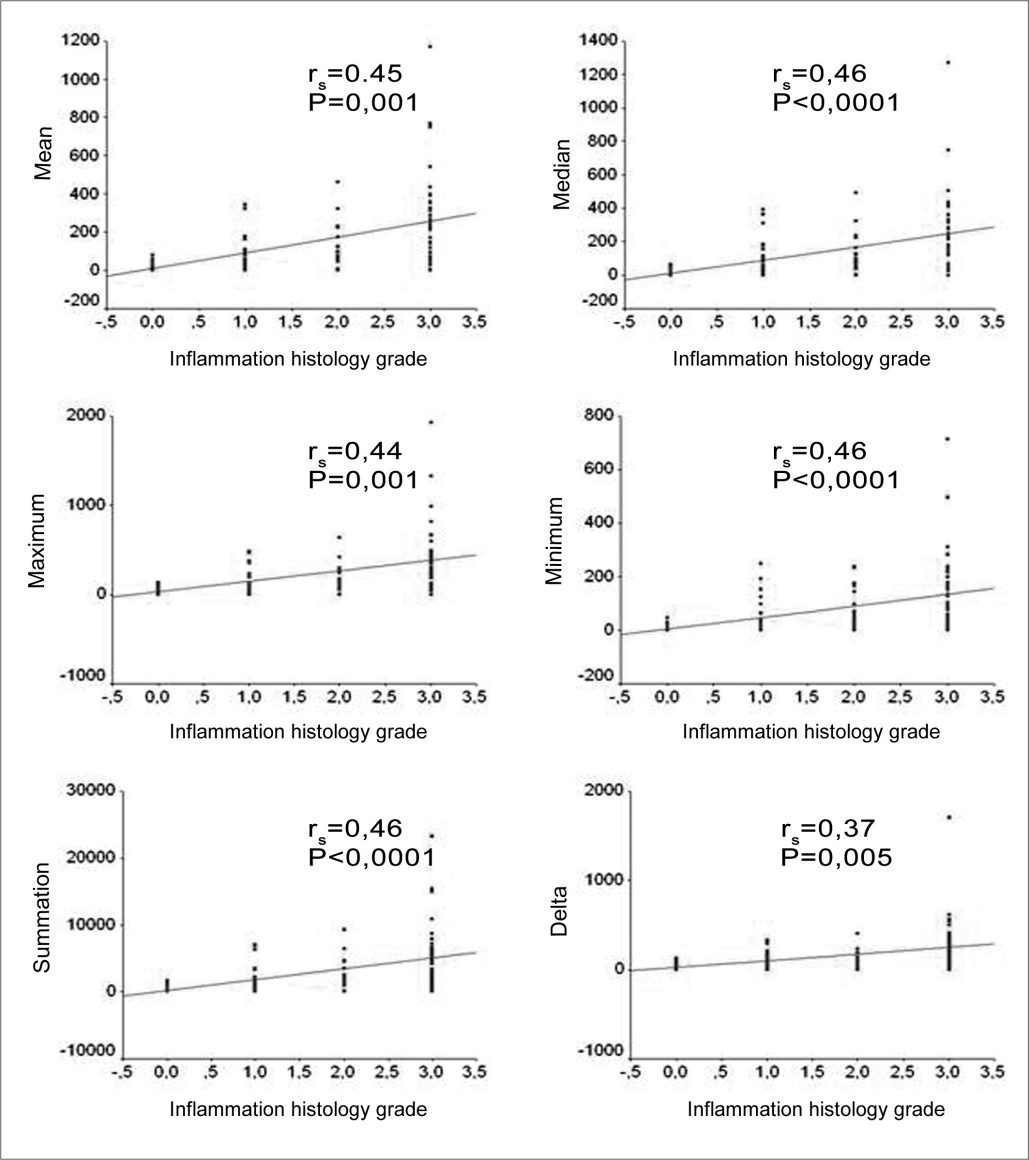
Charts showing the correlations between the inflammation histologic grade and the mean, median, minimum value, maximum value, delta and summation variables.

When we analyzed the degree of perimatrix inflammation, seen at the light microscope, 60% of the samples were classified as moderate to intense. When we applied the Spearman correlations coefficient between the degree of inflammation and the variables summarized in the perimatrix thickness measure (mean, median, maximum value, minimum value, delta and total) we found significant correlations, with magnitudes varying from moderate to large. Figure 5 shows the correlation graphs.

**Figure 6:**
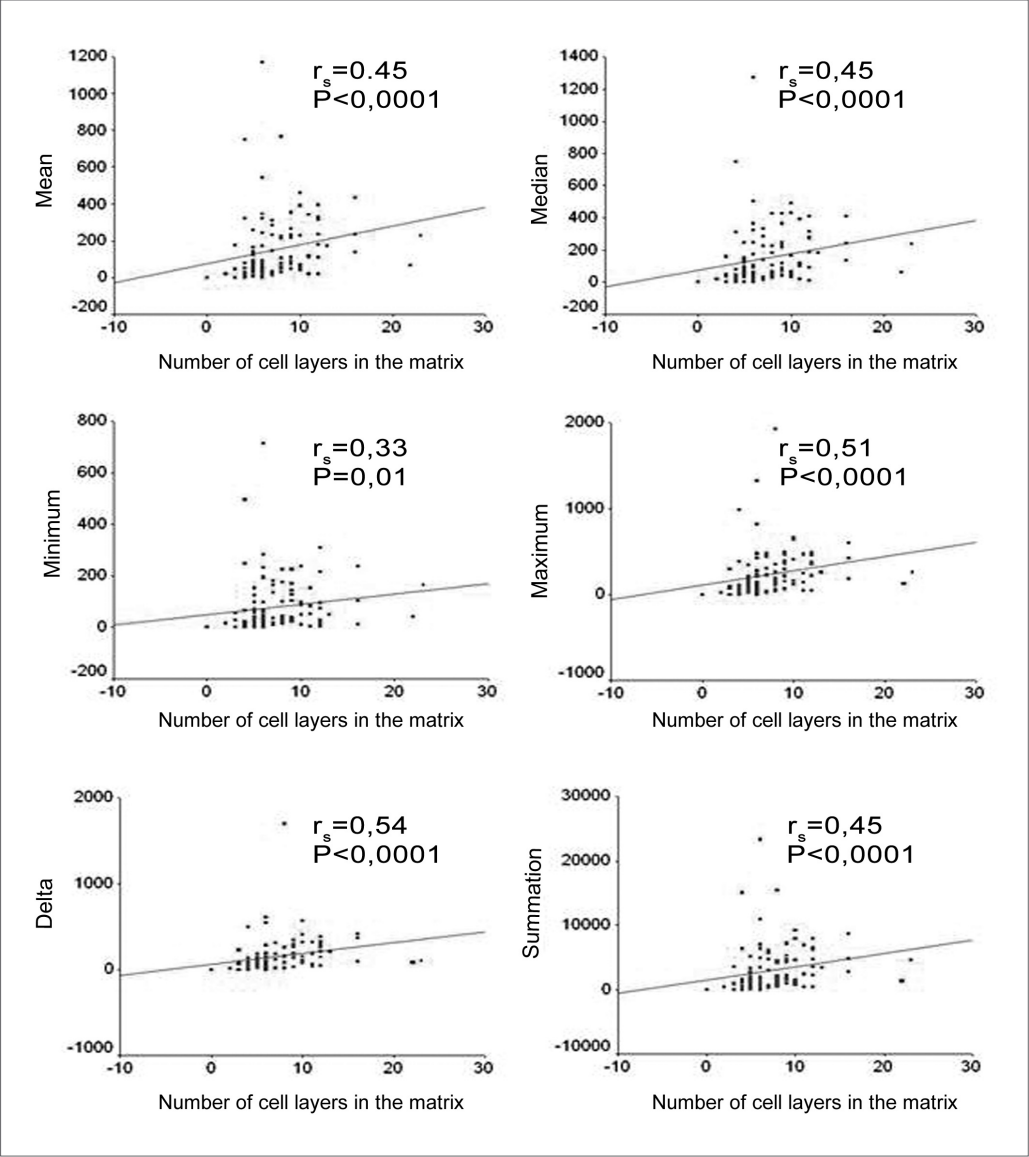
Charts showing the correlation between the average number of cell layers in the matrix and the mean, median, minimum value, maximum value, delta and summation variables.

When we correlated the epithelial cell layers average number with the perimatrix thickness we found correlations that were from moderate to intense, in all the variables tested (Figures 6).

**Figure 7:**
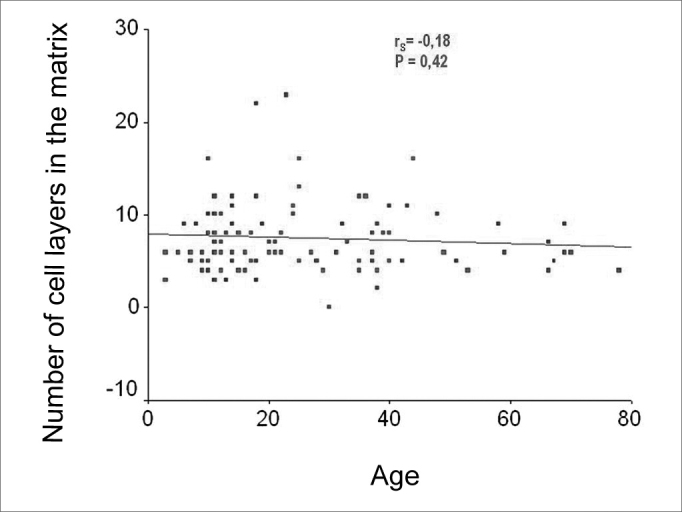
Chart showing the linear correlation between age and the average number of epithelial cell layers in the matrix.

**Figure 8:**
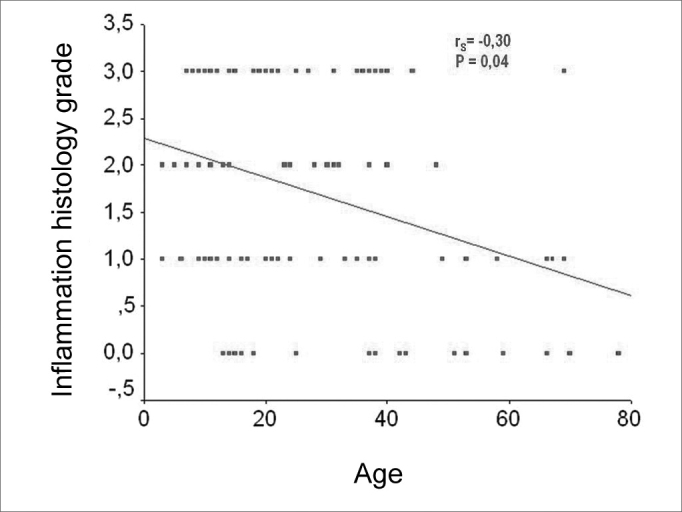
Chart showing the linear correlation between age and the histology grade of inflammation on the perimatrix.

As we used the Person coefficient with the mean number of matrix cell layers and the patient's age, we did not find any correlation. Notwithstanding, the same coefficient has correlations between age and the perimatrix inflammation histology grade (Figures 7 and 8).

When broken down by age group, the other histologic components assessed - matrix hyperplasia, outlining epithelium, fibrosis and granuloma - also presented distribution rates that were similar in both groups (Table 1).

**Table 1:** Comparison between the adult and pediatric groups as to the cholesteatoma histo-morphologic characteristics

Characteristics	Pediatric Group	Adult Group	P Value
Matrix cell layers	7.9 ± 4.2	8.0 ± 4.1	0.89*
Matrix hyperplasia	16.7%	25.0%	0.41**
Outlining epithelium	26.7%	16.7%	0.32**
Fibrosis	73.3%	63.9%	0.41**
Granuloma	13.3%	16.7%	0.71**

* t test

** c^2^ test

## DISCUSSION

Lim and Saunders^3^ were among the first to present a detailed histology of cholesteatomas. They state that cholesteatomas have a keratinized stratified squamous cell epithelium, with the four layers identical to those of the normal epidermis (in greater quantity than it happens in normal epidermis (basal, spinous, granulosa and cornea), Langerhans cells (in greater quantity than that found in the normal epidermis) and kerato-hyalin granules, they called such epithelium as cholesteatoma matrix. They also observed the presence of a lose connective tissue, with collagen fibers, fibrocytes and inflammatory cells, which was called perimatrix. In most of the cases, the latter was in contact with a layer of squamous or hair cylindrical cells, remains from the middle ear mucosa. In some cases, despite the fact that the perimatrix is absent under light microscopy, it was always present under the electron microscope, very thin, almost without collagen fibers and with calcium carbonate crystals. In the present study we were able to confirm those findings from Lim and Saunders^3^. We found a great variability in the perimatrix thickness of cholesteatomas, both intra and inter-patients of both groups.

**Figure 9:**
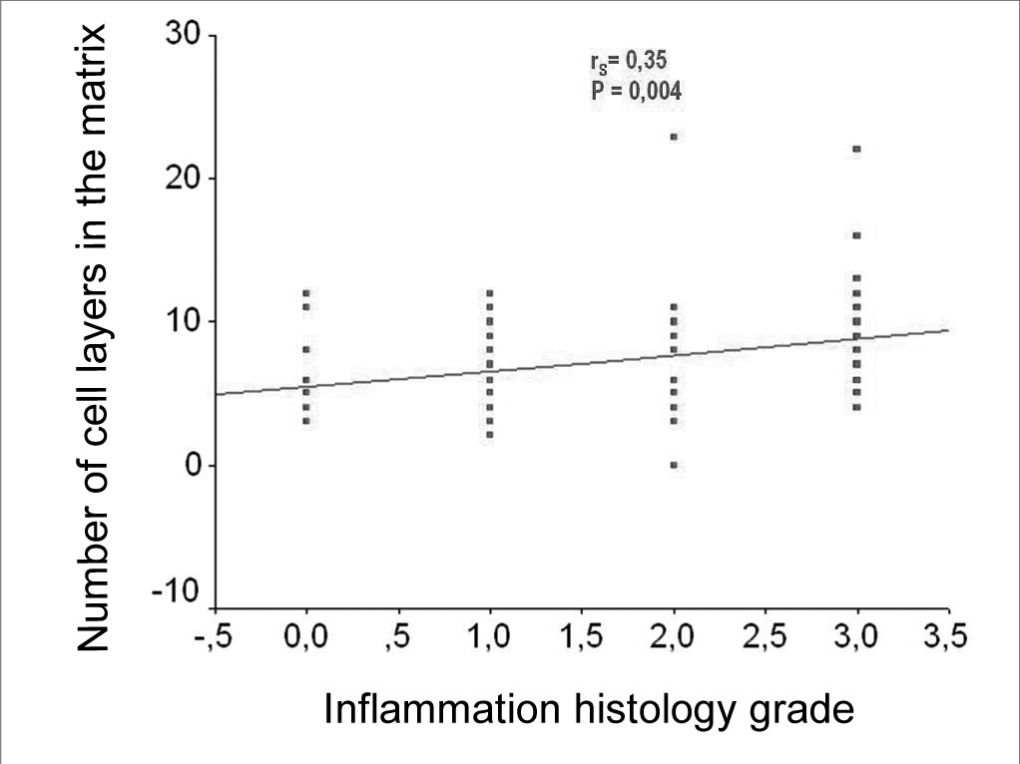
Chart showing a linear correlation between the average number of cell layers on the matrix and the histologic grade of inflammation on the perimatrix.

However, when we compared the cholesteatomas histologically, within the different age ranges (adult and pediatric), we did not find statistically significant differences as to the average number of cell layers on the matrix, in the presence of matrix hyperplasia, in the existence of perimatrix outlining epithelium, and in the presence of perimatrix fibrosis and granuloma. Notwithstanding, through the Pearson correlation coefficient, we found hints that the histological degree of inflammation in the perimatrix reduces with the increase in age. In the same way, the Spearman coefficient shows a tendency to reduce the perimatrix thickness in relation to age increase and surgery.

We can compare the perimatrix to a “battle field”, where the middle ear territory would be at stake. On the one side there is the cholesteatoma; on the other side there is the adjacent tissue of the middle ear. With the cholesteatoma growth, the inflammatory reaction would increase, and consequently would produce more elements of the inflammatory cascade. We believe that it is in the perimatrix, and within the process that happens there, that we have the very aggressiveness of cholesteatomas; thus, based on our findings, we may suggest that the different clinical characteristics of pediatric cholesteatomas would be related to the amount of inflammation they cause.

Besides clues of a greater degree of inflammation in pediatric cholesteatomas, we also found, in this case, a direct correlation, from moderate to intense, between the average number of epithelial cell layers in the matrix with the perimatrix thickness measures. Such fact could indicate that not only the perimatrix is more active in pediatric cholesteatomas, but also that the matrix would have a more active proliferation either current or past. Our findings corroborate the hypothesis from Bujia et al.^12^ who suggested that pediatric cholesteatomas would present a more pronounced proliferative state.

Studies about the impact inflammatory mechanisms have on the CCOM and the role of the perimatrix in the process are necessary in order to unveil these issues, and this should be the next step in this line of research. Although the technique used in the current study is accurate in the measure of the perimatrix thickness, it is not specific in order to detect what element is reduced in its structure. For that, we intend to pursue this line, progressing towards the quantification of the elements that make up the perimatrix of acquired cholesteatomas.

## CONCLUSIONS

In this sample we did not find evidences of differences in the histological components of acquired cholesteatomas in adults and children.

There was an inverse, mild to moderate correlation between the perimatrix size, measured in micrometers, with patient age at the time of the surgery.

The perimatrix degree of inflammation presented moderate to intense correlation with the perimatrix thickness.

The perimatrix inflammation degree presented an inverse correlation with patient's age at the time of the surgery.

The matrix and perimatrix thickness were strongly correlated.
